# Supplementary material for: Coordination Polymers Based on a Biphenyl Tetraphosphonate Linker: Synthesis Control and Photoluminescence
Source: Molecules. 2020 Apr 16;25(8):1835. doi: 10.3390/molecules25081835 (PMC7221988; doi:10.3390/molecules25081835)
Supplement: Supplementary file 1 [file molecules-25-01835-s001.pdf]

Supplementary Materials

Coordination Polymers Based on a Biphenyl Tetraphosphonate Linker: Synthesis Control and Photoluminescence

Ana D. G. Firmino [1,2,†], Ricardo F. Mendes [1,†], Duarte Ananias [1,3], Jéssica S. Barbosa [1,2], João P. C. Tomé [2,4] and Filipe A. Almeida Paz [1,*]

[1] Department of Chemistry, CICECO – Aveiro Institute of Materials, University of Aveiro, 3810-193 Aveiro, Portugal; danielafirmino1@ua.pt (A.D.G.F.); rfmendes@ua.pt (R.F.M.); dananias@ua.pt (D.A.); jessicambarbosa@ua.pt (J.S.B.)

[2] QOPNA and LAQV-REQUIMTE, Department of Chemistry, University of Aveiro, 3810-193 Aveiro, Portugal; jtome@tecnico.ulisboa.pt

[3] Department of Physics, CICECO – Aveiro Institute of Materials, University of Aveiro, 3810-193 Aveiro, Portugal

[4] CQE and Departamento de Engenharia Química Instituto Superior Técnico, Universidade de Lisboa Av. Rovisco Pais, no 1, 1049-001 Lisboa, Portugal

* Correspondence: filipe.paz@ua.pt; Tel.: (+351) 234 401418

† Authors contributed equally to the present work

Table of Contents

1. Experimental Section S3

1.1. General Instrumentation S3

1.2. Reagents and Solvents....................S3

1.3. Synthetic conditions to obtain single-crystals of [$La_4(H_3btp)(H_4btp)(H_5btp)(H_2O)_8$]·$3H_2O$ (2La) S4

1.4. Photoluminescence Spectroscopy S4

2. Additional Crystallographic Data....................S5

3. Characterization Studies S11

3.1. Powder X-ray Diffraction....................S11

3.2. Thermogravimetry S12

3.3. FT-IR Spectroscopy S13

3.4. EDS Mapping....................S15

4. Topological Studies....................S18

5. Photoluminescence Studies S20

6. References....................S21

1. Experimental Section

1.1. General Instrumentation

SEM (scanning electron microscopy) images were acquired using either a Hitachi S4100 field emission gun tungsten filament instrument working at 25 kV or a high-resolution Hitachi SU-70 working at 4 kV. Samples were prepared by deposition on aluminium sample holders followed by carbon coating using an Emitech K950X carbon evaporator. EDS (energy dispersive X-ray spectroscopy) data and SEM mapping images were recorded using the latter microscope working at 15 kV and using either a Bruker Quantax 400 or an Esprit 1.9 EDS microanalysis system.

Thermogravimetric analyses (TGA) were carried out using a Shimadzu TGA 50, from ambient temperature to *ca.* 800 ºC (heating rate of 5 ºC/min) under a continuous stream of air at a flow rate of 20 mL min^{-1}.

Fourier Transform Infrared (FT-IR) spectra (in the range 4000–350 cm^{-1}) were recorded as KBr pellets (2 mg of sample was mixed in a mortar with 200 mg of KBr; purity > 99%, BDH SpectrosoL) using a Bruker Tensor 27 spectrometer by averaging 256 scans at a maximum resolution of 2 cm^{-1}.

Elemental analyses for C and H were performed with a Truspec Micro CHNS 630-200-200 elemental analyzer at the Department of Chemistry, University of Aveiro. Analysis parameters: sample amount between 1 and 2 mg; combustion furnace temperature = 1075 ºC; afterburner temperature = 850 ºC. Detection method: carbon and hydrogen; infrared absorption. Analysis time = 4 minutes. Required gasses: carrier, helium; combustion, oxygen; pneumatic, compressed air.

Routine Powder X-Ray Diffraction (PXRD) data for all prepared materials were collected at ambient temperature on a Empyrean PANalytical diffractometer (Cu K$\alpha_{1,2}$ X-radiation, λ_1 = 1.540598 Å; λ_2 = 1.544426 Å), equipped with an PIXcel 1D detector and a flat-plate sample holder in a Bragg–Brentano para-focusing optics configuration (45 kV, 40 mA). Intensity data were collected by the step-counting method (step 0.01º), in continuous mode, in the *ca.* 3.5 ≤ 2θº ≤ 50º range.

Variable-temperature powder X-ray diffraction data were collected on an PANalyticalX'Pert Powder diffractometer Cu $K_{a1,2}$ X-radiation (λ_1 = 1.540598 Å; λ_2 = 1.544426 Å), equipped with an PIXcel 1D detector, and a flat-plate sample holder in a Bragg–Brentano para-focusing optics configuration (40 kV, 50 mA), and a high-temperature Anton Paar HKL 16 chamber controlled by an Anton Paar 100 TCU unit. Intensity data were collected in the continuous mode (*ca.* 100 seconds data acquisition) in the angular range *ca.* 3.5 ≤ 2θ° ≤ 50 (step 0.01º).

1.2. Reagents and Solvents

[1,1′-Biphenyl]-3,3′,5,5′-tetrayltetrakis(phosphonic acid) (**H_8btp**) was prepared as previously reported.[1-2] Chemicals, including solvents, were readily available from commercial sources and were used as received without further purification: europium(III) chloride hexahydrate ($EuCl_3{\cdot}6H_2O$, 99.9%, Sigma-Aldrich); cerium(III) chloride heptahydrate ($CeCl_3{\cdot}7H_2O$, 99.9%, Sigma-Aldrich); praseodymium(III) chloride hydrate ($PrCl_3{\cdot}xH_2O$, 99.9%, Sigma-Aldrich); neodymium(III) chloride hexahydrate ($NdCl_3{\cdot}6H_2O$, 99.9%, Sigma-Aldrich); gadolinium chloride hexahydrate ($GdCl_3{\cdot}6H_2O$, 99.999%, Sigma-Aldrich); hydrochloric acid (HCl, 37%, José Manuel Gomes dos Santos Lta.); methanol (CH_4O, >99.8%, Fluka or Chromasolv for HPLC, ≥99.9%, Sigma-Aldrich).

1.3. Synthetic conditions to obtain single-crystals of [La_4(H_3btp)(H_4btp)(H_5btp)(H_2O)$_8$]·3H_2O (2La)

A reactive mixture composed of lanthanum(III) chloride heptahydrate ($LaCl_3{\cdot}7H_2O$, 0.211 mmol; 0.0783 g) and [1,1′-biphenyl]-3,3′,5,5′-tetrayltetrakis(phosphonic acid) (H_8btp, 0.0527 mmol; 0.025 g), with an overall molar ratio of approximately 1:4 (H_8btp : La^{3+}), was prepared in a mixture of distilled water and HCl (6 M) (9 mL of distilled water; 1 mL of HCl solution). The mixture was kept under constant magnetic stirring in open air and ambient temperature for approximately 15 min. The resulting homogeneous suspension was transferred to a Teflon-lined Parr Instrument reaction vessel and placed inside a MMM Venticell oven. The heating program used was that described previously. The resulting crystals were used as-synthesized for single-crystal X-ray diffraction studies, where the phase [La_4(H_3btp)(H_4btp)(H_5btp)(H_2O)$_8$]·3H_2O was identified. The remaining crystals were recovered by vacuum filtration, washed with abundant amounts of distilled water and dried at ambient temperature. PXRD indicated a mixture of phases: [La_4(H_3btp)(H_4btp)(H_5btp)(H_2O)$_8$]·3H_2O is present along with a slight unidentified contamination.

1.4. Photoluminescence Spectroscopy

The emission and excitation spectra were recorded at ambient-temperature and 12 K using a Fluorolog®-3Horiba Scientific (Model FL3-2T) spectroscope, with a modular double grating excitation spectrometer (fitted with a 1200 grooves/mm grating blazed at 330 nm) and a TRIAX 320 single emission monochromator (fitted with a 1200 grooves/mm grating blazed at 500 nm, reciprocal linear density of 2.6 nm.mm-1), coupled to a R928 Hamamatsu photomultiplier, using the front face

acquisition mode. The excitation source was a 450 W Xe arc lamp. The emission spectra were corrected for detection and optical spectral response of the spectrofluorimeter and the excitation spectra were corrected for the spectral distribution of the lamp intensity using a photodiode reference detector. Time-resolved measurements have been carried out using a 1934D3 phosphorimeter coupled to the Fluorolog®-3, and a Xe-Hg flash lamp (6 μs/pulse half width and 20-30 μs tail) was used as the excitation source. The low temperature measurements (12 K) were performed using a helium-closed cycle cryostat with vacuum system measuring *ca.* 5×10^{-6} mbar and a Lakeshore 330 auto-tuning temperature controller with a resistance heater.

2. Additional Crystallographic Data

Table S1. Selected bond lengths (in Å) and angles (in degrees) for the metallic coordination environments present in $[Eu_7(H_5btp)_4(H_{5.5}btp)_2(H_6btp)_2(H_2O)_{12}]\cdot 23.5H_2O\cdot MeOH$ (**1Eu**).[a]

Eu1–O22	2.221(7)	Eu3–O28^{iii}	2.270(7)
Eu1–O22	2.221(7)	Eu3–O15	2.295(7)
Eu1–O43^{i}	2.245(7)	Eu3–O12	2.297(8)
Eu1–O43	2.246(7)	Eu3–O1^{iii}	2.305(7)
Eu1–O34^{i}	2.277(6)	Eu3–O18^{iii}	2.341(8)
Eu1–O34	2.277(6)	Eu3–O5W	2.436(8)
Eu2–O3W	2.535(16)	Eu3–O4W	2.472(8)
Eu2–O31	2.278(6)	Eu4–O13	2.267(7)
Eu2–O32^{ii}	2.294(7)	Eu4–O29^{iii}	2.283(7)
Eu2–O7	2.301(7)	Eu4–O37	2.303(7)
Eu2–O19	2.332(6)	Eu4–O7W	2.465(8)
Eu2–O41^{ii}	2.352(7)	Eu4–O6W	2.451(8)
Eu2–O1W	2.447(7)	Eu4–O25	2.310(8)
Eu2–O2W	2.470(13)	Eu4–O47^{iii}	2.310(8)
O22–Eu1–O22^{i}	180.0(3)	O15–Eu3–O12	88.5(3)
O22–Eu1–O43^{i}	88.1(3)	O28^{iii} –Eu3–O1^{iii}	90.4(3)
O22^{i} –Eu1–O43^{i}	91.9(3)	O28^{iii} –Eu3–O15	89.7(3)
O22–Eu1–O43	91.9(3)	O28^{iii} –Eu3–O12	138.5(3)
O22^{i} –Eu1–O43	88.1(3)	O15–Eu3–O18^{iii}	82.3(2)
O43^{i} –Eu1–O43	180.0	O12–Eu3–O18^{iii}	138.9(3)
O22–Eu1–O34^{i}	90.7(2)	O1^{iii} –Eu3–O18^{iii}	91.0(3)
O22^{i} –Eu1–O34^{i}	89.3(2)	O28^{iii} –Eu3–O5W	71.1(3)
O43^{i} –Eu1–O34^{i}	86.4(3)	O15–Eu3–O5W	99.9(3)
O43–Eu1–O34^{i}	93.6(3)	O12–Eu3–O4W	70.8(3)
O22–Eu1–O34	89.3(2)	O1^{iii} –Eu3–O4W	80.0(3)
O22^{i} –Eu1–O34	90.7(2)	O18^{iii} –Eu3–O4W	70.6(3)
O43^{i} –Eu1–O34	93.6(3)	O5W–Eu3–O4W	135.3(3)
O43–Eu1–O34	86.4(3)	O15–Eu3–O1^{iii}	173.2(2)
O34^{i} –Eu1–O34	180.0	O12–Eu3–O1^{iii}	95.9(3)
O7–Eu2–O19	87.6(2)	O28^{iii} –Eu3–O18^{iii}	81.6(3)
O31–Eu2–O41^{ii}	141.2(2)	O12–Eu3–O5W	68.4(3)
O32^{ii} –Eu2–O41^{ii}	88.9(2)	O1^{iii} –Eu3–O5W	86.6(3)
O7–Eu2–O41^{ii}	85.2(2)	O18^{iii} –Eu3–O5W	152.6(3)
O19–Eu2–O41^{ii}	140.3(2)	O28^{iii} –Eu3–O4W	150.4(3)
O31–Eu2–O2W	151.1(3)	O15–Eu3–O4W	96.7(3)

O32^{ii} –Eu2–O2W	95.2(4)	O13–Eu4–O29^{iii}	89.6(3)
O7–Eu2–O2W	81.6(4)	O13–Eu4–O47^{iii}	136.9(3)
O19–Eu2–O2W	73.8(3)	O29^{iii} –Eu4–O47^{iii}	89.7(3)
O41^{ii} –Eu2–O2W	66.5(3)	O37–Eu4–O47^{iii}	90.1(3)
O41^{ii} –Eu2–O3W	74.7(4)	O25–Eu4–O47^{iii}	143.0(3)
O1W–Eu2–O3W	140.6(4)	O13–Eu4–O6W	151.0(3)
O31–Eu2–O32^{ii}	93.7(3)	O29^{iii} –Eu4–O7W	97.8(3)
O31–Eu2–O7	91.5(3)	O37–Eu4–O7W	83.8(3)
O32^{ii} –Eu2–O7	174.0(3)	O25–Eu4–O7W	148.7(3)
O31–Eu2–O19	77.9(2)	O13–Eu4–O37	91.8(3)
O32^{ii} –Eu2–O19	96.4(2)	O29^{iii} –Eu4–O37	178.2(3)
O31–Eu2–O1W	71.0(2)	O13–Eu4–O25	80.1(3)
O32^{ii} –Eu2–O1W	88.7(2)	O29^{iii} –Eu4–O25	92.1(3)
O7–Eu2–O1W	90.1(2)	O37–Eu4–O25	87.0(3)
O19–Eu2–O1W	148.7(2)	O29^{iii} –Eu4–O6W	92.3(3)
O41^{ii} –Eu2–O1W	70.4(2)	O37–Eu4–O6W	85.9(3)
O1W–Eu2–O2W	136.6(3)	O25–Eu4–O6W	70.8(3)
O31–Eu2–O3W	142.6(4)	O47^{iii} –Eu4–O6W	72.1(3)
O32^{ii} –Eu2–O3W	72.9(4)	O13–Eu4–O7W	70.3(3)
O7–Eu2–O3W	104.6(4)	O47^{iii} –Eu4–O7W	67.1(3)
O19–Eu2–O3W	69.5(4)	O6W–Eu4–O7W	137.8(3)
O2W–Eu2–O3W	23.7(4)		

[a] Symmetry transformations used to generate equivalent atoms: (i) –x, 1–y, 1–z; (ii) –x, 1–y, –z; (iii) x, 3/2–y, –1/2+z.

Table S2. Hydrogen bonding geometry (distances in Å and angles in degrees) of $[Eu_7(H_5btp)_4(H_{5.5}btp)_2(H_6btp)_2(H_2O)_{12}]\cdot 23.5H_2O\cdot MeOH$ (**1Eu**). [a]

D–H···A	$d_{D\cdots A}$	<(DHA)
Phosphonate-Phosphonate Interactions		
O45–H45···O30	2.661(12)	125
O2–H2···O48^{v}	2.496(13)	128
O33–H33···O49^{ii}	3.24(3)	134
O14–H14···O9	2.544(11)	138
O49–H49···O38^{ii}	2.94(3)	142
O11–H11···O39^{v}	2.648(13)	143
O33–H33···O38	2.749(11)	146
O17–H17···O(10	2.499(12)	149
O24–H24···O5^{vi}	2.569(11)	150
O4–H4···O4^{vi}	3.20(2)	152
O20–H20···O6	2.557(11)	152
O36–H36···O40	2.584(10)	155
O48–H48···O2^{x}	2.496(13)	157
O16–H16···O26	2.897(17)	158
O42–H42···O6^{x}	3.061(15)	159
O30–H30···O23	3.263(13)	162
O44–H44···O24^{i}	2.916(13)	162
O38–H38···O33	2.749(11)	171
O23–H23···O30	3.263(13)	176
Phosphonate-Water Interactions		
O27–H27···O22W^{ix}	2.51(4)	111
O23–H23···O13W^{vi}	2.97(2)	112
O30–H30···O13W^{vi}	2.90(2)	117
O49–H49···O3W	3.04(3)	120
O16–H16···O8W^{viii}	3.067(18)	128
O27–H27···O23W^{ix}	2.46(4)	143
O8–H8···O10W	3.007(8)	152
O9–H9···O11W^{vii}	2.677(16)	154
O26–H26···O8W^{viii}	2.745(16)	155

[a] Symmetry transformations used to generate equivalent atoms: (i) -*x*, 1-*y*, 1-*z*, (ii) -*x*, 1-*y*, -*z*, (v) 1+*x*, *y*, *z*, (vi) 1-*x*, 1-*y*, 1-*z*, (vii) 1-*x*, 1-*y*, -*z*, (viii) 1-*x*, 1/2+*y*, 1/2-*z* (ix) -*x*, 1/2+*y*, 1/2-*z*, (x) -1+*x*, *y*, *z*.

Table S3. Selected bond lengths (in Å) and angles (in degrees) for the coordination environment present in $[La_4(H_3btp)(H_4btp)(H_5btp)(H_2O)_8]\cdot 3H_2O$ (**2La**).

La1–O32^{i}	2.387(5)	La3–O29	2.834(6)
La1–O4^{ii}	2.393(5)	La4–O1	2.346(5)
La1–O15^{iii}	2.456(4)	La4–O8^{vi}	2.387(5)
La1–O14	2.504(4)	La4–O33^{vii}	2.425(4)
La1–O28	2.508(5)	La4–O20^{viii}	2.438(5)
La1–O1W	2.626(5)	La4–O18^{ix}	2.453(5)
La1–O3W	2.641(5)	La4–O26	2.477(5)
La1–O2W	2.727(5)	La4–O8W	2.714(5)
La2–O10^{ii}	2.387(5)	O2W–La3^{iii}	2.789(5)
La2–O21^{iv}	2.389(5)	O3–La3^{vii}	2.378(5)
La2–O22	2.407(5)	O4–La1^{vii}	2.393(5)
La2–O12^{v}	2.433(5)	O8–La4^{x}	2.387(5)
La2–O6W	2.554(6)	O10–La2^{vii}	2.387(5)
La2–O4W	2.566(5)	O12–La2^{v}	2.433(5)
La2–O34	2.715(5)	O14–La3^{iii}	2.490(4)
La2–O5W	2.851(6)	O15–La1^{iii}	2.456(4)
La3–O3^{ii}	2.378(5)	O15–La3^{iii}	2.761(5)
La3–O13	2.457(5)	O18–La4^{xi}	2.453(5)
La3–O36^{i}	2.473(5)	O20–La4^{xii}	2.438(4)
La3–O14^{iii}	2.490(4)	O21–La2^{iv}	2.389(5)
La3–O28	2.592(5)	O32–La1^{xiii}	2.387(5)
La3–O7W	2.631(5)	O33–La4^{ii}	2.425(4)
La3–O15^{iii}	2.761(5)	O36–La3^{xiii}	2.473(5)
La3–O2W^{iii}	2.789(5)		
O32^{i}–La1–O4^{ii}	137.94(16)	O10^{ii}–La2–O5W	137.90(18)
O32^{i}–La1–O15^{iii}	84.22(15)	O21^{iv}–La2–O5W	68.21(16)
O4^{ii}–La1–O15^{iii}	132.33(15)	O22–La2–O5W	119.01(17)
O32^{i}–La1–O14	140.65(16)	O12^{v}–La2–O5W	132.42(18)
O4^{ii}–La1–O14	74.78(16)	O6W–La2–O5W	66.5(2)
O15^{iii}–La1–O14	81.42(14)	O4W–La2–O5W	67.79(17)
O32^{i}–La1–O28	98.68(17)	O34–La2–O5W	65.35(16)
O4^{ii}–La1–O28	79.59(17)	O3^{ii}–La3–O13	90.87(17)
O15^{iii}–La1–O28	71.13(15)	O3^{ii}–La3–O36^{i}	121.62(17)

O14–La1–O28	110.77(15)	O13–La3–O36^{i}	145.06(16)
O32^{i}–La1–O1W	67.37(16)	O3^{ii}–La3–O14^{iii}	135.02(16)
O4^{ii}–La1–O1W	70.66(16)	O36^{i}–La3–O7W	70.46(17)
O15^{iii}–La1–O1W	139.04(16)	O14^{iii}–La3–O7W	79.38(19)
O14–La1–O1W	138.94(16)	O28–La3–O7W	152.38(19)
O28–La1–O1W	84.22(16)	O3^{ii}–La3–O15^{iii}	159.40(15)
O32^{i}–La1–O3W	94.82(17)	O13–La3–O15^{iii}	72.01(15)
O4^{ii}–La1–O3W	72.46(17)	O36^{i}–La3–O15^{iii}	73.43(15)
O15^{iii}–La1–O3W	137.26(16)	O14^{iii}–La3–O15^{iii}	55.35(13)
O14–La1–O3W	72.55(16)	O28–La3–O15^{iii}	65.18(14)
O28–La1–O3W	149.94(16)	O7W–La3–O15^{iii}	124.42(17)
O1W–La1–O3W	76.40(17)	O3^{ii}–La3–O2W^{iii}	73.75(17)
O32^{i}–La1–O2W	77.98(16)	O13–La3–O2W^{iii}	68.12(16)
O4^{ii}–La1–O2W	124.91(18)	O36^{i}–La3–O2W^{iii}	129.42(15)
O15^{iii}–La1–O2W	75.64(15)	O14^{iii}–La3–O2W^{iii}	62.25(15)
O14–La1–O2W	63.02(15)	O28–La3–O2W^{iii}	137.35(16)
O28–La1–O2W	146.76(15)	O7W–La3–O2W^{iii}	67.94(18)
O1W–La1–O2W	122.90(16)	O15^{iii}–La3–O2W^{iii}	108.69(14)
O3W–La1–O2W	62.53(16)	O3^{ii}–La3–O29	71.22(18)
O10^{ii}–La2–O21^{iv}	110.60(18)	O13–La3–O29	115.50(15)
O10^{ii}–La2–O22	82.96(17)	O36^{i}–La3–O29	69.36(16)
O21^{iv}–La2–O22	151.22(18)	O14^{iii}–La3–O29	150.68(16)
O10^{ii}–La2–O12^{v}	83.67(18)	O28–La3–O29	53.61(15)
O21^{iv}–La2–O12^{v}	76.46(17)	O7W–La3–O29	99.75(19)
O22–La2–O12^{v}	80.18(16)	O15^{iii}–La3–O29	105.32(14)
O10^{ii}–La2–O6W	152.8(2)	O2W^{iii}–La3–O29	144.80(16)
O21^{iv}–La2–O6W	88.4(2)	O1–La4–O8^{vi}	156.16(18)
O22–La2–O6W	71.80(19)	O1–La4–O33^{vii}	90.08(17)
O12^{v}–La2–O6W	82.1(2)	O8^{vi}–La4–O33^{vii}	106.03(17)
O10^{ii}–La2–O4W	90.25(17)	O1–La4–O20^{viii}	89.66(18)
O21^{iv}–La2–O4W	132.13(16)	O8^{vi}–La4–O20^{viii}	78.99(17)
O22–La2–O4W	70.49(16)	O33^{vii}–La4–O20^{viii}	72.43(16)
O12^{v}–La2–O4W	150.58(17)	O1–La4–O18^{ix}	106.04(18)
O6W–La2–O4W	90.8(2)	O8^{vi}–La4–O18^{ix}	95.45(17)
O10^{ii}–La2–O34	74.35(16)	O33^{vii}–La4–O18^{ix}	73.72(16)
O21^{iv}–La2–O34	71.58(17)	O20^{viii}–La4–O18^{ix}	142.53(17)

O22–La2–O34	137.19(16)	O1–La4–O26	91.47(18)
O12^{v}–La2–O34	131.04(16)	O8^{vi}–La4–O26	85.78(18)
O6W–La2–O34	131.79(19)	O33^{vii}–La4–O26	143.76(17)
O4W–La2–O34	73.75(16)	O20^{viii}–La4–O26	143.77(17)
O13–La3–O14^{iii}	81.63(15)	O18^{ix}–La4–O26	71.07(17)
O36^{i}–La3–O14^{iii}	83.07(15)	O1–La4–O8W	73.66(17)
O3^{ii}–La3–O28	98.71(16)	O8^{vi}–La4–O8W	82.63(17)
O13–La3–O28	70.14(16)	O33^{vii}–La4–O8W	136.84(15)
O36^{i}–La3–O28	90.83(15)	O20^{viii}–La4–O8W	67.89(16)
O14^{iii}–La3–O28	119.47(15)	O18^{ix}–La4–O8W	148.81(15)
O3^{ii}–La3–O7W	75.87(19)	O26–La4–O8W	77.74(16)
O13–La3–O7W	136.05(19)		

[a] Symmetry transformations used to generate equivalent atoms: (i) -x+1, y-1/2, -z+3/2; (ii) x+1, y, z; (iii) -x+1, -y, -z+1; (iv) -x+1, -y+1, -z+1; (v) -x, -y+1, -z+1; (vi) x+1, -y+1/2, z+1/2; (vii) x-1, y, z; (viii) x-1, -y+1/2, z+1/2; (ix) x, -y+1/2, z+1/2; (x) x-1, -y+1/2, z-1/2; (xi) x, -y+1/2, z-1/2; (xii) x+1, -y+1/2, z-1/2; (xiii) -x+1, y+1/2, -z+3/2.

Table S4. Hydrogen bonding geometry (distances in Å and angles in degrees) of $[La_4(H_3btp)(H_4btp)(H_5btp)(H_2O)_8]\cdot 3H_2O$ (**2La**).

**D–H···A**	$d_{D\cdots A}$	**<(DHA)**
Phosphonate-Phosphonate Interactions		
O24–H24···O27^{xi}	2.842(8)	111
O30–H30···O23^{ix}	2.562(7)	140
O6–H6···O25^{vii}	2.529(8)	155
O19–H19···O7^{xvi}	2.704(7)	163
O31–H31···O11^{ii}	2.734(7)	163
O11–H11···O23^{v}	2.647(7)	166
O27–H27···O24^{ix}	2.842(8)	166
O16–H16···O5^{ii}	2.521(7)	170
O2–H2···O25	2.771(7)	172
Phosphonate-Water Interactions		
O35–H35···O4W	2.803(7)	131

O9–H9···O6W[xv]	3.013(8)	157
O7–H7···O10W[xv]	2.553(9)	161

[a] Symmetry transformations used to generate equivalent atoms: (ii) x+1, y, z; (v) -x, -y+1, -z+1; (vii) x-1, y, z; (ix) x, -y+1/2, z+1/2; (xi) x, -y+1/2, z-1/2; (xiv) -x-1, -y+1, -z+1; (xv) x+2, y, z.

3. Characterization Studies

3.1. Powder X-ray Diffraction

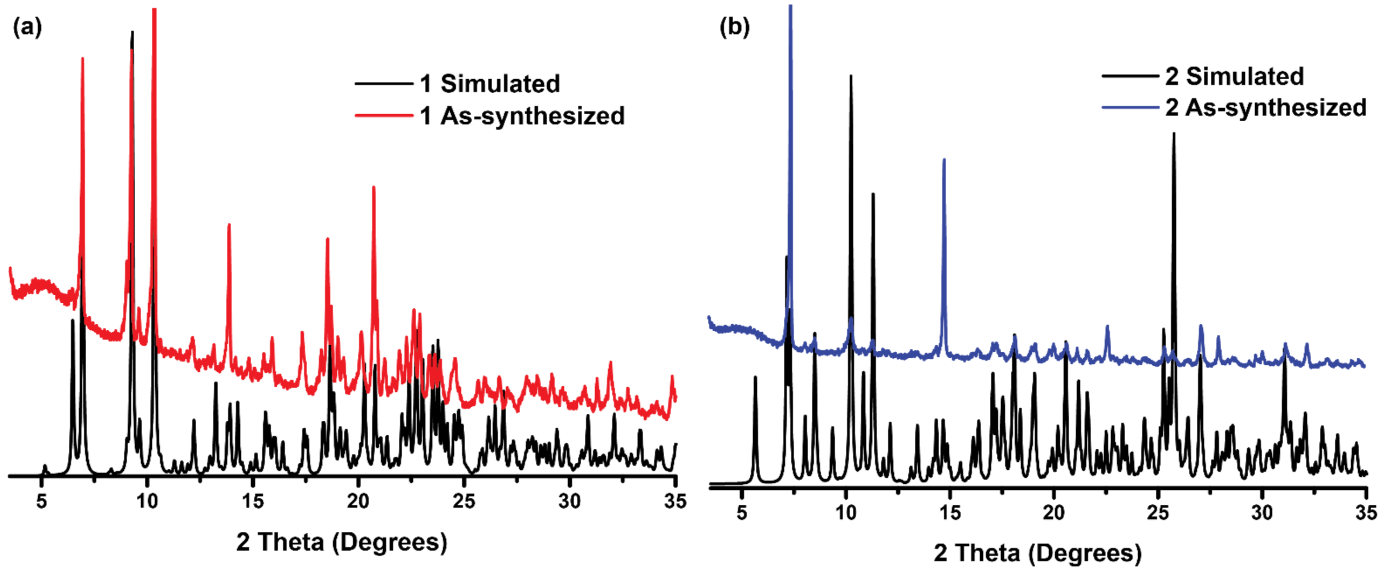

Figure S1 - Powder X-ray diffraction patterns simulated and experimental of (**a**) $Ln_7(H_5btp)_4(H_{5.5}btp)_2(H_6btp)_2(H_2O)_{12}]\cdot 23.5H_2O\cdot MeOH$ [where Ln^{3+} = Eu^{3+} (**1Eu**) and Gd^{3+} (**1Gd**)] and (**b**) $[Ln_4(H_3btp)(H_4btp)(H_5btp)(H_2O)_8]\cdot 3H_2O$ [where Ln^{3+} = Ce^{3+} (**2Ce**), Pr^{3+} (**2Pr**), and Nd^{3+} (**2Nd**)].

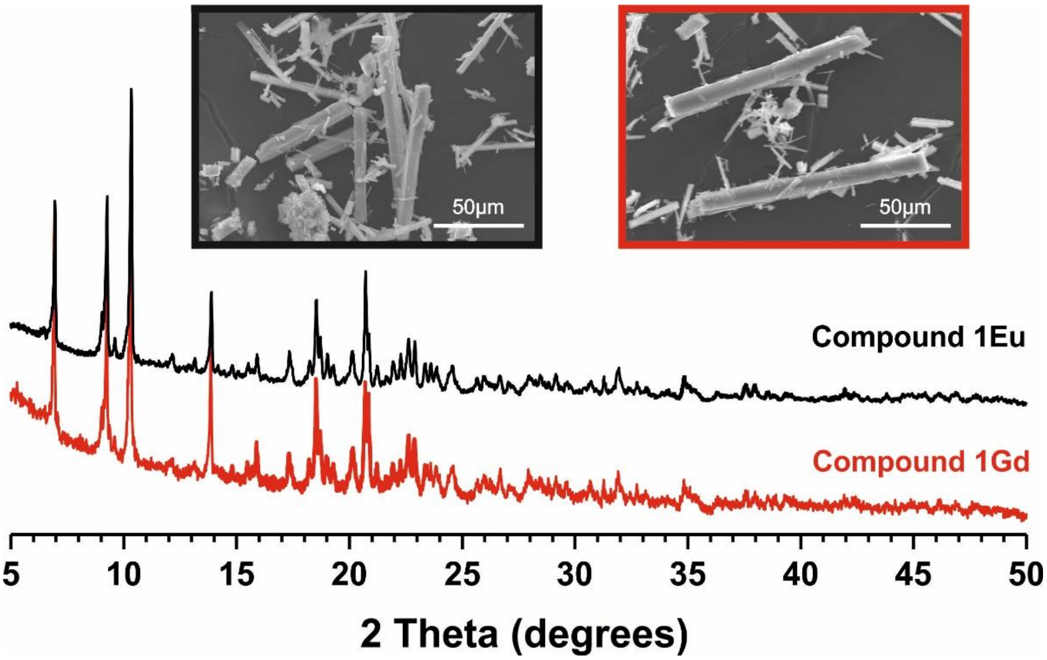

Figure S2. Powder X-ray diffraction patterns and SEM images of bulk materials $Ln_7(H_5btp)_4(H_{5.5}btp)_2(H_6btp)_2(H_2O)_{12}]\cdot 23.5H_2O\cdot MeOH$ [where Ln^{3+} = Eu^{3+} (**1Eu**) and Gd^{3+} (**1Gd**)].

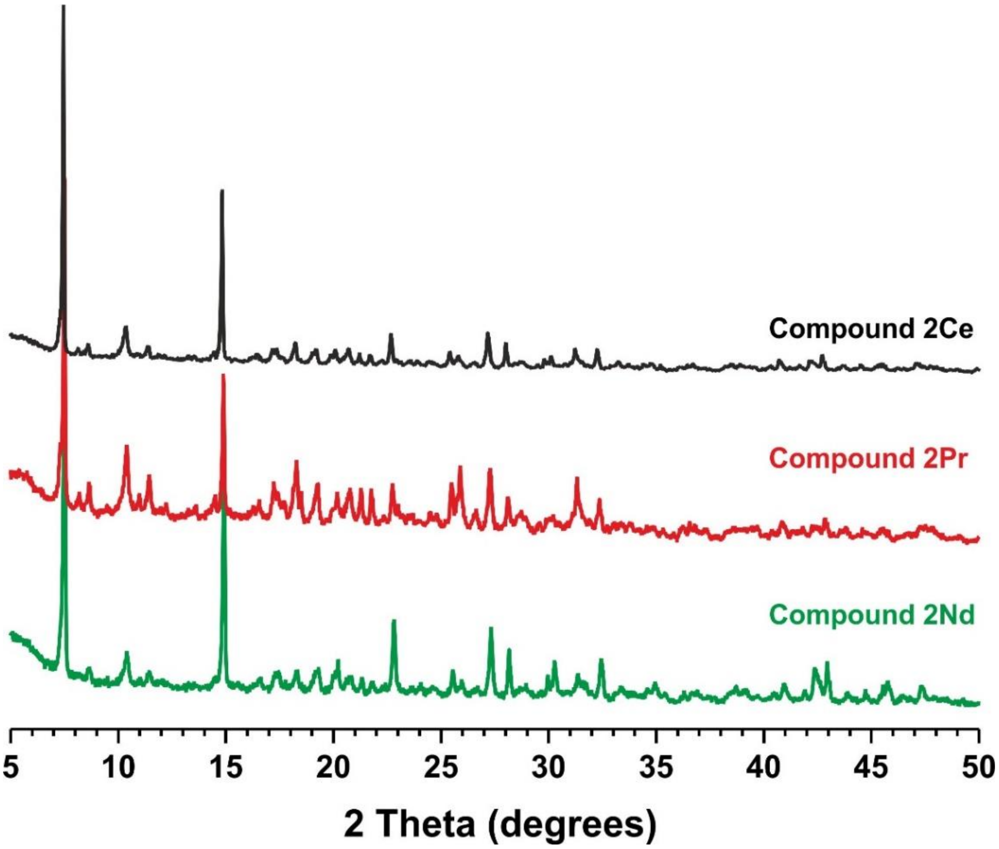

Figure S3. Powder X-ray diffraction patterns of bulk materials $[Ln_4(H_3btp)(H_4btp)(H_5btp)(H_2O)_8]\cdot 3H_2O$ [where Ln^{3+} = Ce^{3+} (**2Ce**), Pr^{3+} (**2Pr**), and Nd^{3+} (**2Nd**)].

3.2. Thermogravimetry

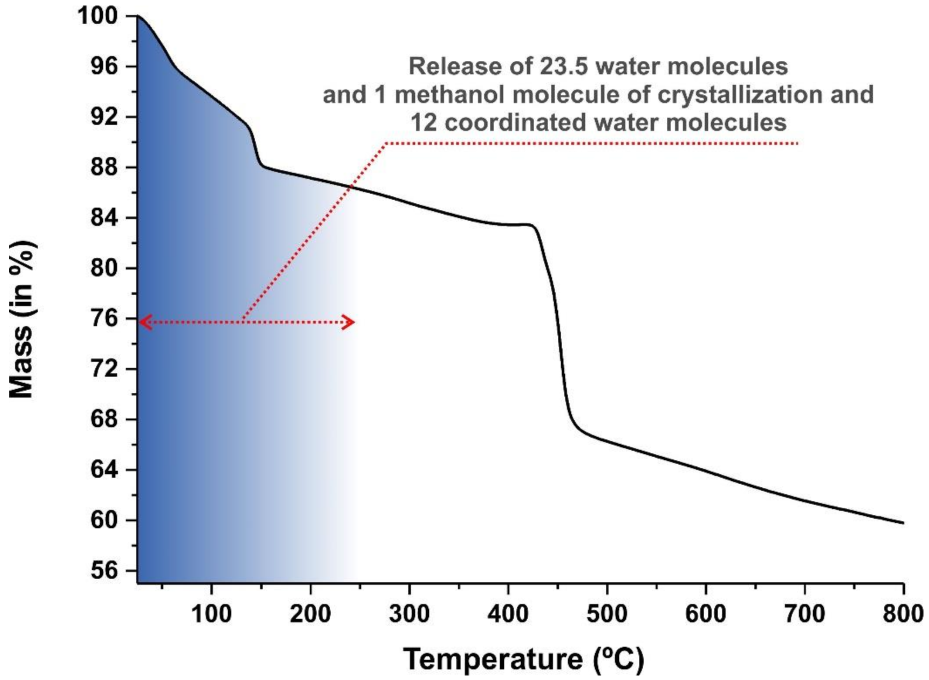

Figure S4. Thermogram of $[Gd_7(H_5btp)_4(H_{5.5}btp)_2(H_6btp)_2(H_2O)_{12}]\cdot 23.5H_2O\cdot MeOH$ (**1Gd**) collected between ambient temperature and *ca.* 800 ºC.

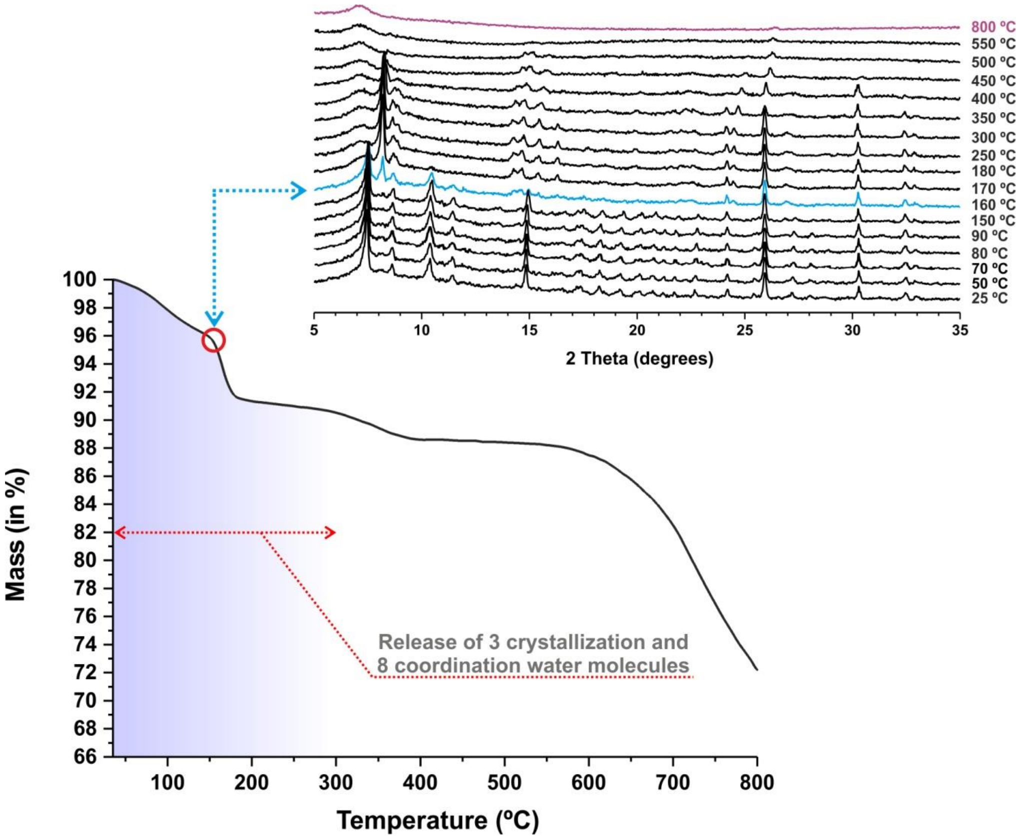

Figure S5. Thermogram of [$Ce_4(H_3btp)(H_4btp)(H_5btp)(H_2O)_8$]·$3H_2O$ (**2Ce**) collected between ambient temperature and *ca.* 800 ºC. (*Inset)* Thermodiffractometry data of the same material collected in the same temperature range.

3.3. FT-IR Spectroscopy

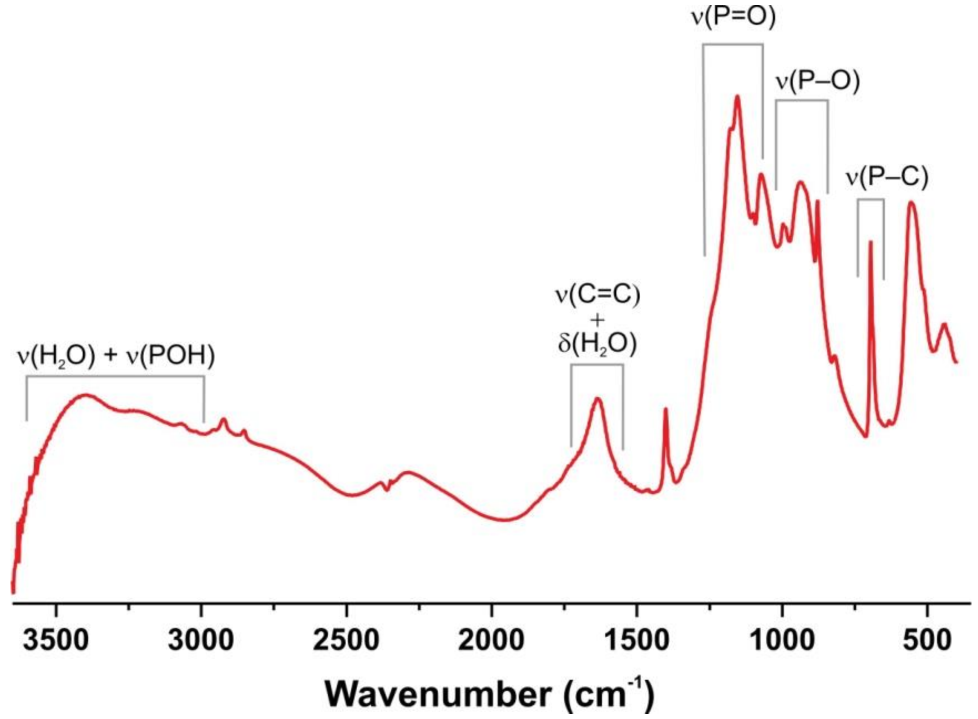

Figure S6. FT-IR spectra of [$Eu_7(H_5btp)_4(H_{5.5}btp)_2(H_6btp)_2(H_2O)_{12}$]·$23.5H_2O$·MeOH (**1Eu**).

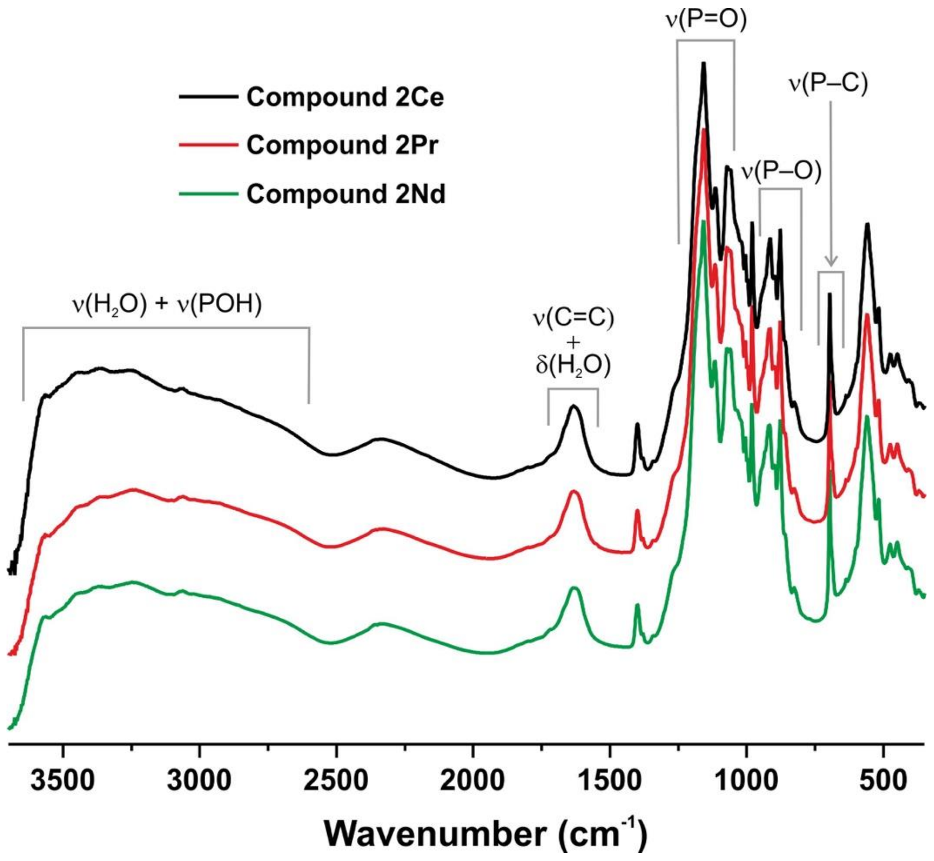

Figure S7. FT-IR spectra of the isotypical family of compounds $[Ln_4(H_3btp)(H_4btp)(H_5btp)(H_2O)_8]\cdot3H_2O$ [where Ln^{3+} = Ce^{3+} (**2Ce**), Pr^{3+} (**2Pr**), and Nd^{3+} (**2Nd**)].

3.4. EDS Mapping

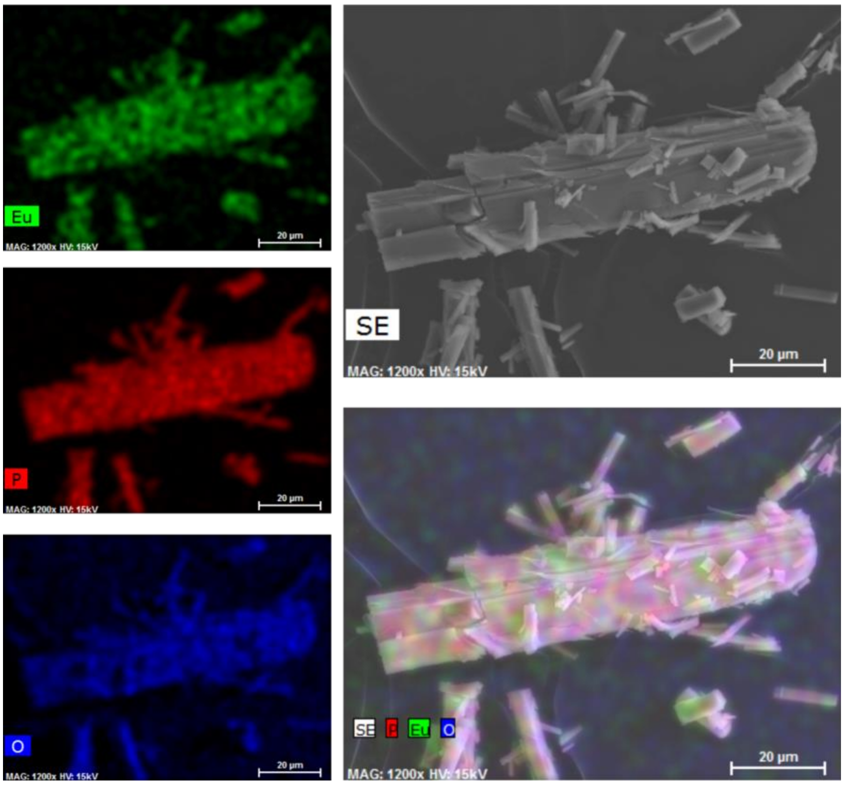

Figure S8. EDS mapping of the [$Eu_7(H_5btp)_4(H_{5.5}btp)_2(H_6btp)_2(H_2O)_{12}$]·23.5$H_2O$·MeOH (**1Eu**) bulk material. Calculated Eu : P ratio of 1.0 : 4.6.

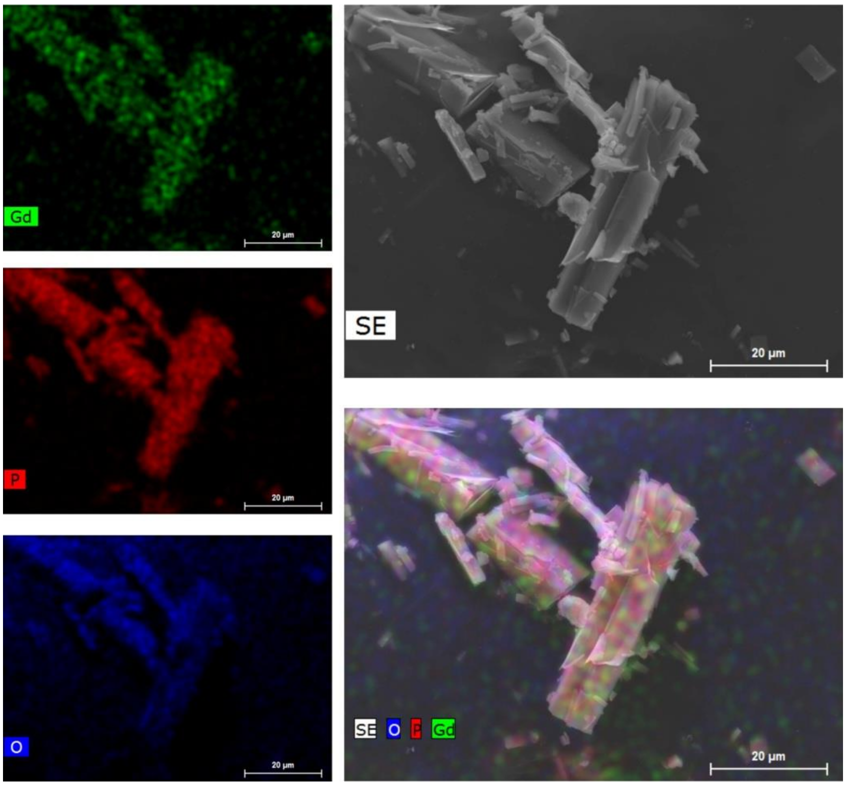

Figure S9. EDS mapping of the [$Gd_7(H_5btp)_4(H_{5.5}btp)_2(H_6btp)_2(H_2O)_{12}$]·23.5$H_2O$·MeOH (**1Gd**) bulk material. Calculated Gd:P ratio of 1.0:4.2.

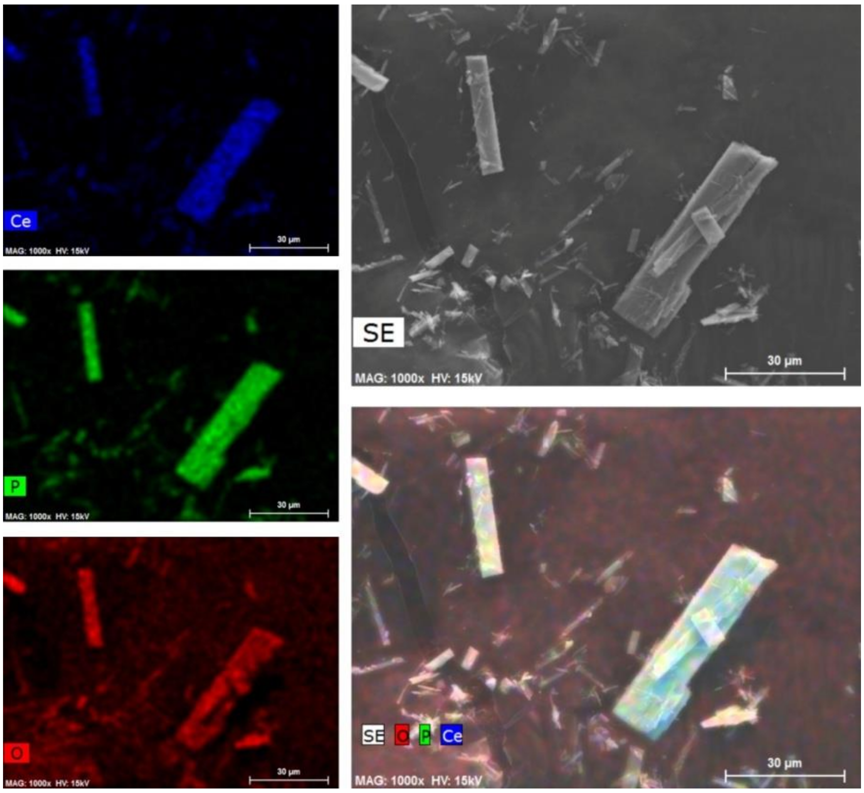

Figure S10. EDS mapping of a representative portion of the $[Ce_4(H_3btp)(H_4btp)(H_5btp)(H_2O)_8]\cdot 3H_2O$ (**2Ce**) bulk material. Calculated Ce:P ratio of 1.0:2.6.

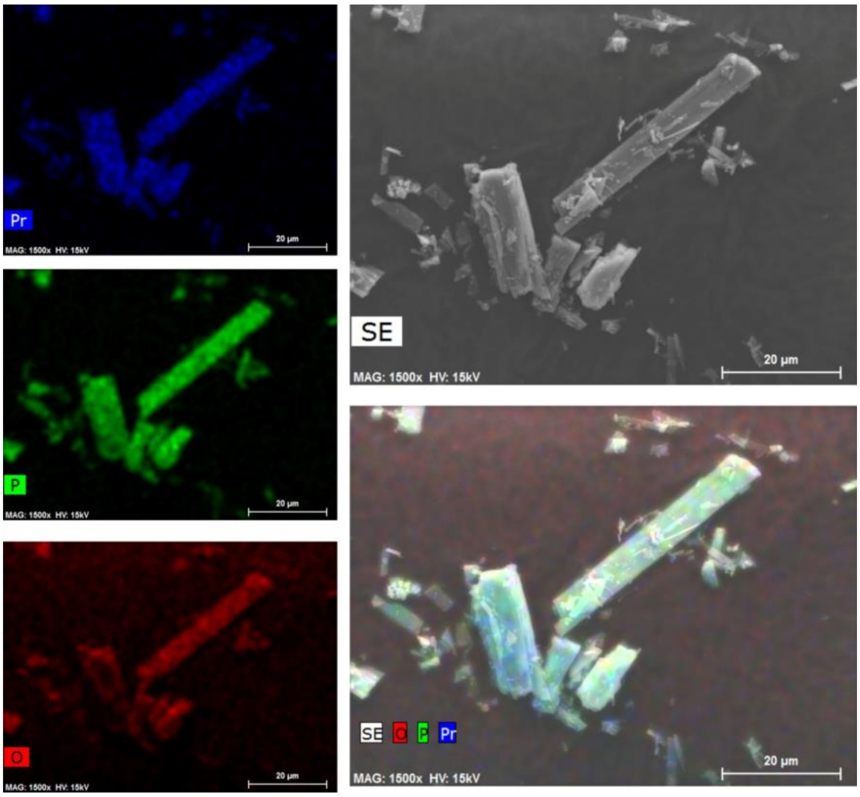

Figure S11. EDS mapping of a representative portion of the $[Pr_4(H_3btp)(H_4btp)(H_5btp)(H_2O)_8]\cdot 3H_2O$ (**2Pr**) bulk material. Calculated Pr:P ratio of 1.0:2.7.

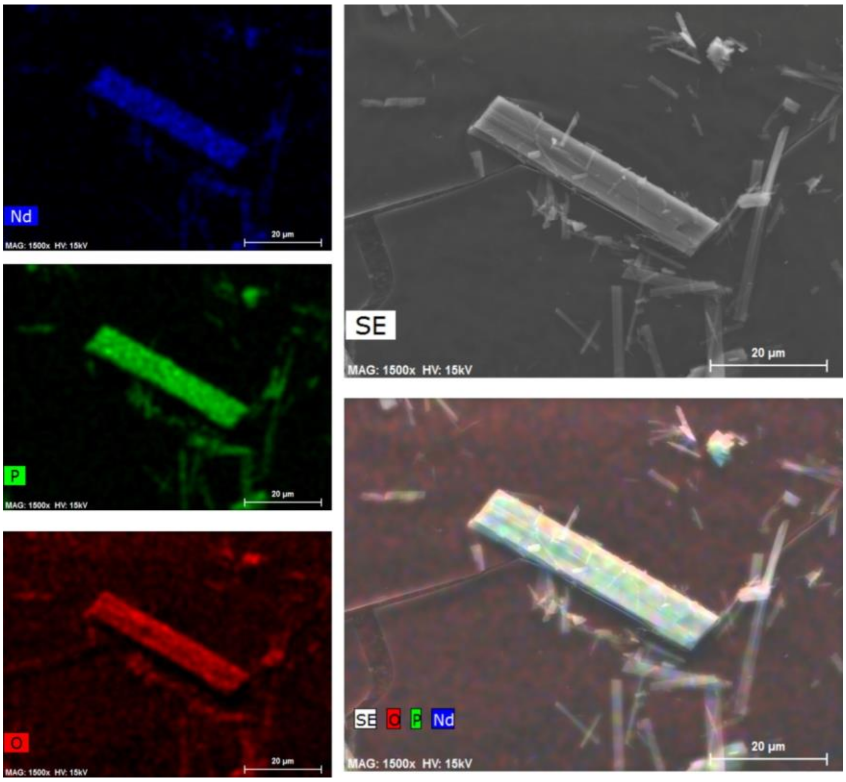

Figure S12. EDS mapping of a representative portion of the [$Nd_4(H_3btp)(H_4btp)(H_5btp)(H_2O)_8$]·$3H_2O$ (**2Nd**) bulk material. Calculated Nd:P ratio of 1.0:2.7.

4. Topological Studies

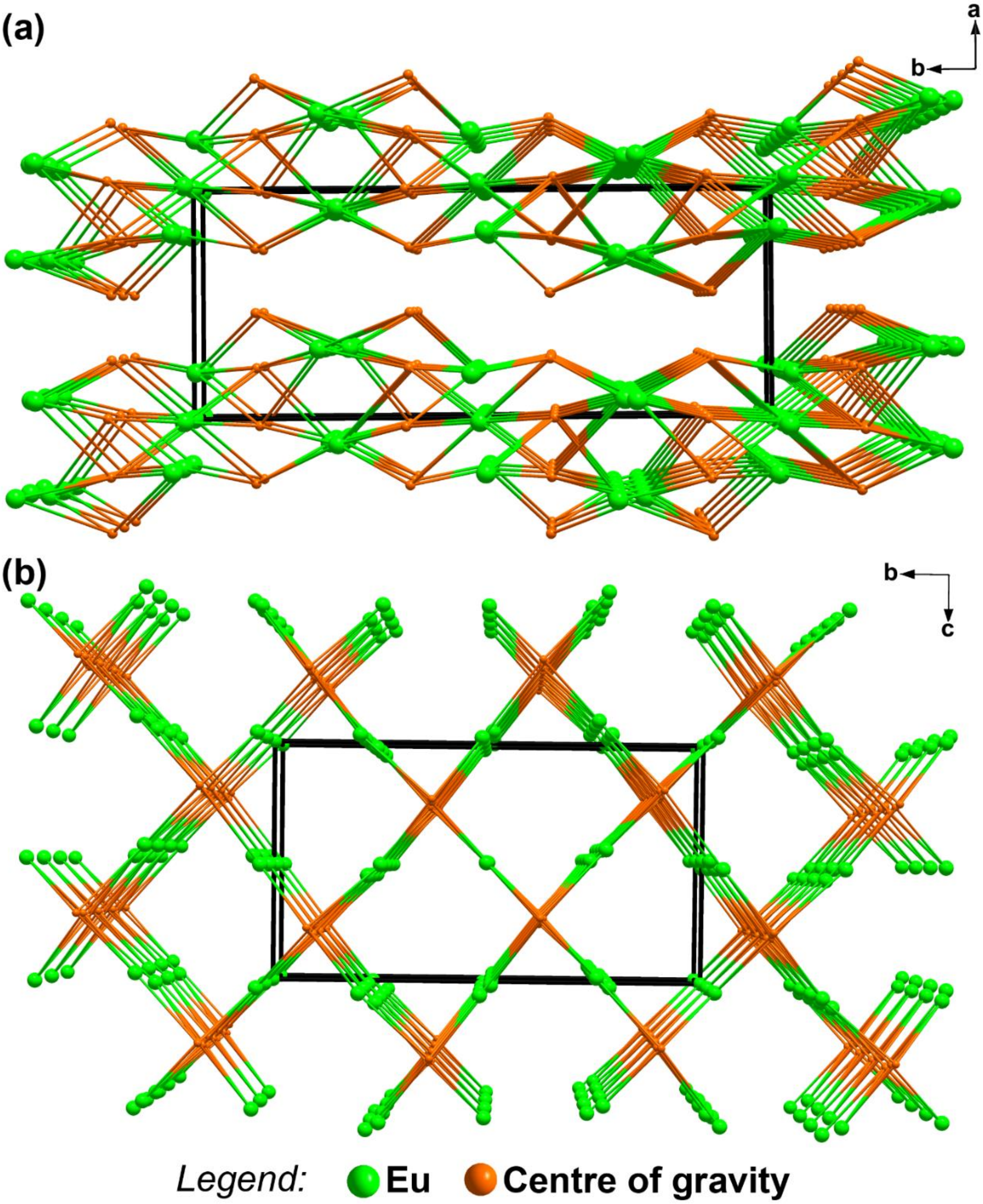

Figure S13. Topological representation of the $[Eu_7(H_5btp)_4(H_{5.5}btp)_2(H_6btp)_2(H_2O)_{12}]\cdot 23.5H_2O\cdot MeOH$ (**1Eu**) compound as an octanodal 3,4,5,5,5,5,6,6-connected network with total point symbol $\{4^{13}\cdot 6^2\}_2\{4^3\}_2\{4^5\cdot 6^3\cdot 8^2\}_6\{4^6\cdot 8^8\cdot 10\}\{4^6\}_2\{4^9\cdot 6\}_2$.

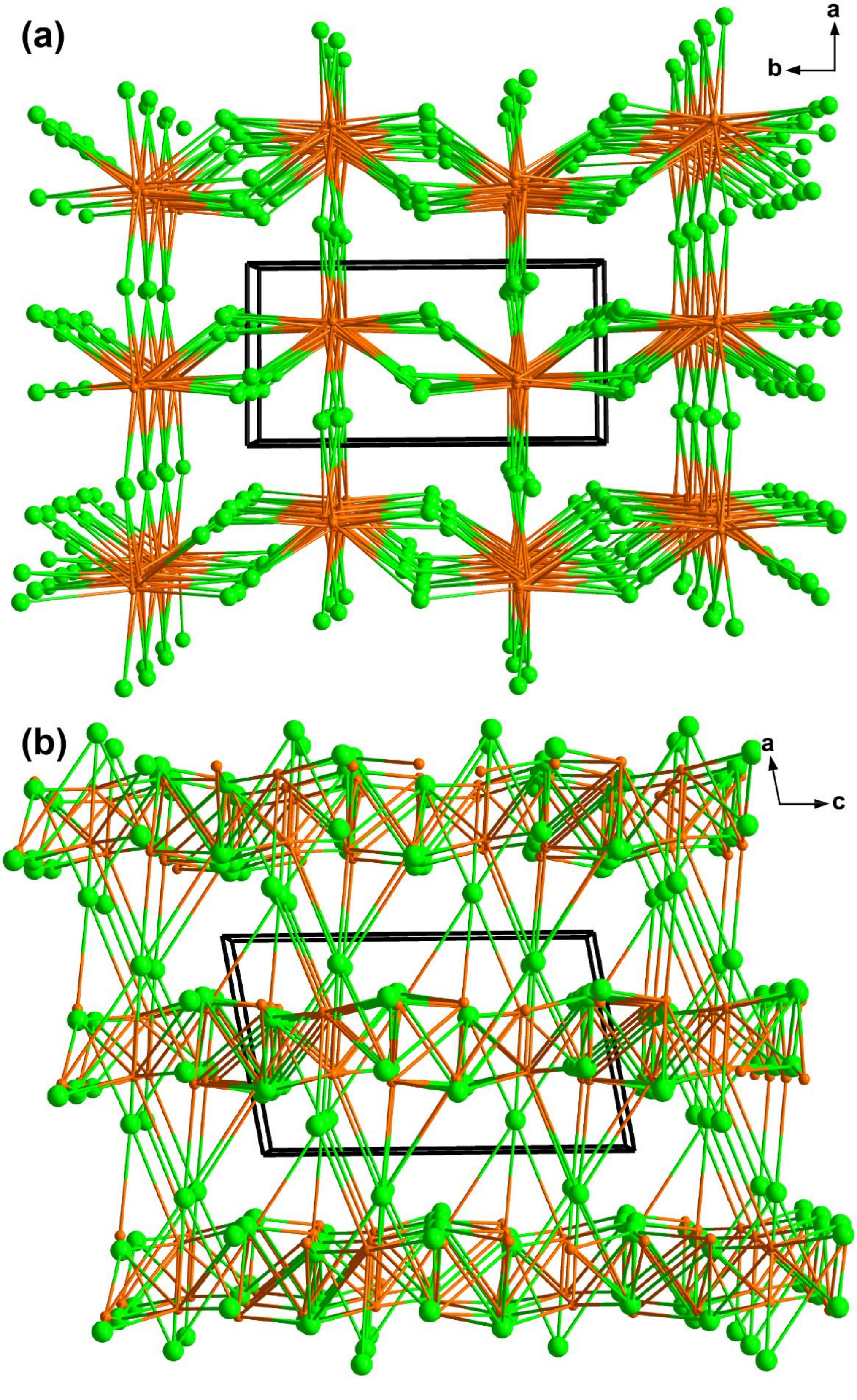

Figure S14. Topological representation of the $[Ln_4(H_3btp)(H_4btp)(H_5btp)(H_2O)_8]\cdot 3H_2O$ (**2La**) compound as a hexanodal 5,6,6,7,7,8-connected network with total point symbol $\{3\cdot4^{12}\cdot6^{2}\}\{3^{2}\cdot4^{10}\cdot6^{9}\}\{3^{4}\cdot4^{16}\cdot5^{6}\cdot6^{2}\}\{3^{7}\cdot4^{11}\cdot5^{3}\}_2\{4^{4}\cdot5\cdot6\cdot8^{9}\}\{4^{8}\cdot6^{2}\}$.

5. Photoluminescence Studies

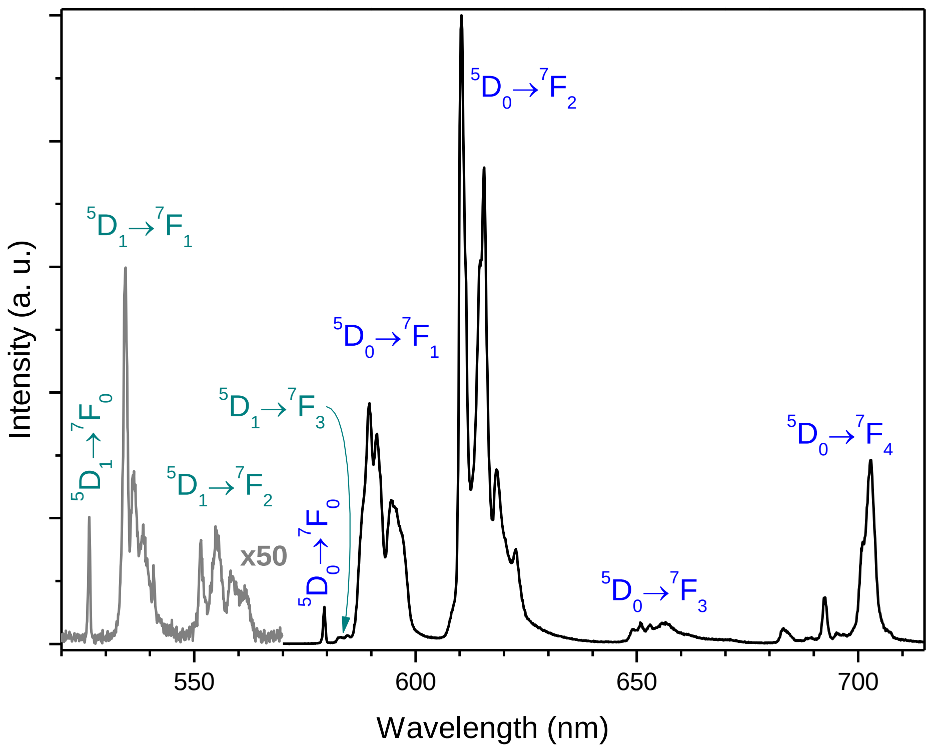

Figure S15. Emission spectrum of $Eu_7(H_5btp)_4(H_{5.5}btp)_2(H_6btp)_2(H_2O)_{12}]\cdot 23.5H_2O\cdot MeOH$ (**1Eu**), recorded at at 12 K, with the excitation fixed at 310 nm.

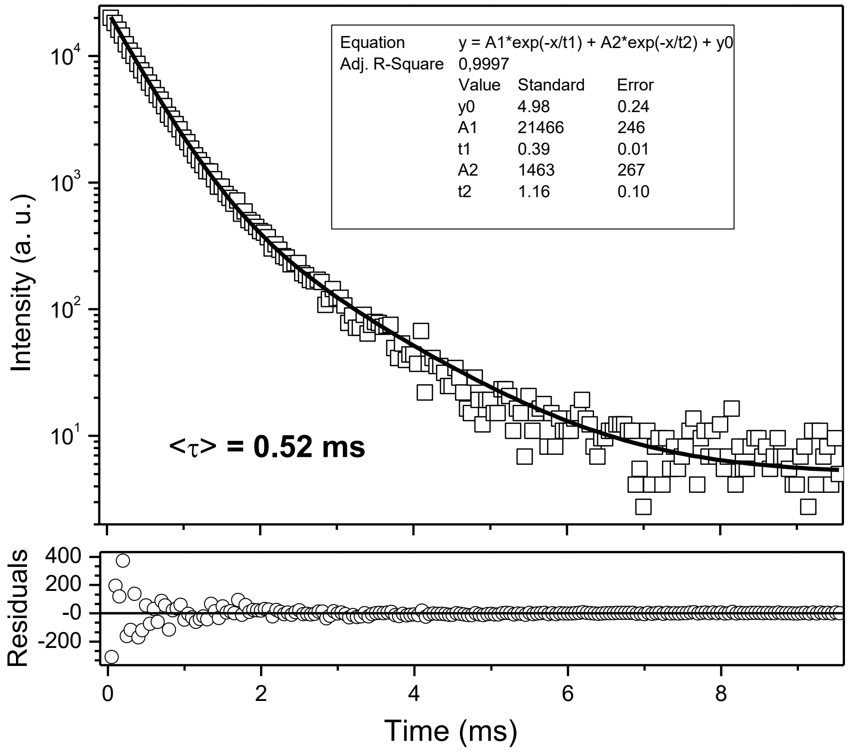

Figure S16. $^5D_0$ decay curves of $[Eu_7(H_5btp)_4(H_{5.5}btp)_2(H_6btp)_2(H_2O)_{12}]\cdot 23.5H_2O\cdot MeOH$ (**1Eu**), acquired at ambient temperature, fitted with a second order exponential decay function, y = y_0+A_1×exp(-x/) + A_2×exp(-x/ $_2$). The average lifetime, ⟨τ⟩, was defined as $(A_1\tau_1^2 + A_2\tau_2^2)/(A_1\tau_1 + A_2\tau_2)$. The emission was monitored at 610.5 nm and the excitation was fixed at 393 nm.

6. References

1. A. D. G. Firmino, R. F. Mendes, D. Ananias, S. M. F. Vilela, L. D. Carlos, J. P. C. Tomé, J. Rocha and F. A. A. Paz. *Inorg. Chim. Acta* **2017**, *455*, 584–594.
2. A. D. G. Firmino, R. F. Mendes, M. M. Antunes, P. C. Barbosa, S. M. F. Vilela, A. A. Valente, F. M. L. Figueiredo, J. P. C. Tomé and F. A. A. Paz, *Inorg. Chem.* **2017**, ***56***, 1193–1208.
